# The Thrombopoietin Receptor, MPL, Is a Therapeutic Target of Opportunity in the MPN

**DOI:** 10.3389/fonc.2021.641613

**Published:** 2021-03-10

**Authors:** Jerry L. Spivak, Alison R. Moliterno

**Affiliations:** Hematology Division, Department of Medicine, Johns Hopkins University School of Medicine Baltimore, Baltimore, MD, United States

**Keywords:** myeloproliferative neoplasms, hematopoietic stem cells, thrombopoietin receptor, thrombopoietin, polycythemia vera, *JAK2* V617F, transgenic mice

## Abstract

The myeloproliferative neoplasms, polycythemia vera, essential thrombocytosis and primary myelofibrosis share driver mutations that either activate the thrombopoietin receptor, MPL, or indirectly activate it through mutations in the gene for JAK2, its cognate tyrosine kinase. Paradoxically, although the myeloproliferative neoplasms are classified as neoplasms because they are clonal hematopoietic stem cell disorders, the mutations affecting MPL or JAK2 are gain-of-function, resulting in increased production of normal erythrocytes, myeloid cells and platelets. Constitutive JAK2 activation provides the basis for the shared clinical features of the myeloproliferative neoplasms. A second molecular abnormality, impaired posttranslational processing of MPL is also shared by these disorders but has not received the recognition it deserves. This abnormality is important because MPL is the only hematopoietic growth factor receptor expressed in hematopoietic stem cells; because MPL is a proto-oncogene; because impaired MPL processing results in chronic elevation of plasma thrombopoietin, and since these diseases involve normal hematopoietic stem cells, they have proven resistant to therapies used in other myeloid neoplasms. We hypothesize that MPL offers a selective therapeutic target in the myeloproliferative neoplasms since impaired MPL processing is unique to the involved stem cells, while MPL is required for hematopoietic stem cell survival and quiescent in their bone marrow niches. In this review, we will discuss myeloproliferative neoplasm hematopoietic stem cell pathophysiology in the context of the behavior of MPL and its ligand thrombopoietin and the ability of thrombopoietin gene deletion to abrogate the disease phenotype *in vivo* in a JAK2 V617 transgenic mouse model of PV.

## Introduction

The myeloproliferative neoplasms (MPNs), polycythemia vera (PV), essential thrombocytosis (ET), and primary myelofibrosis (PMF), are hematopoietic stem cell (HSC) disorders that express mutations activating JAK2 (1–4), the cognate tyrosine kinase for the type 1 homodimeric hematopoietic growth factor receptors for erythropoietin (EPO) and thrombopoietin (THPO) (5), and the granulocyte-colony stimulating factor receptor as well (6). Although these disorders are genetically distinct (7, 8) (9), have different natural histories (10), different complications and require different therapies (11), they exhibit significant clinical phenotypic mimicry, including overproduction of one or more blood cell types alone or together, extramedullary hematopoiesis (EMH) and transformation to myelofibrosis or to acute leukemia (12). Some of these abnormalities can be directly linked to inappropriate constitutive JAK2 activation, which results in committed hematopoietic progenitor cell (HPC) hypersensitivity to hematopoietic growth factors or complete hematopoietic growth factor-independence, depending on whether the *JAK2* mutation is heterozygous or homozygous in the HPC (13). Others cannot, including failure of *JAK2* V617F to enhance MPN HSC bone marrow pool size (14), resistance of MPN HSC to tyrosine kinase inhibitors (15) premature release of mutated CD34+ HSC from the bone marrow (16), myelofibrosis or the clinical presentation of PV and PMF as isolated thrombocytosis, suggesting that pathways other than constitutive JAK2 activation are involved.

The MPNs are clonal HSC disorders and the thrombopoietin receptor, MPL, is the only hematopoietic growth factor expressed in these cells. In addition to its role in signal transduction, JAK2 chaperones MPL cell-surface expression and ensures its stability there (17). THPO maintains HSC survival (18) and quiescence within the bone marrow osteoblastic niche (19, 20) and is required for megakaryocytic progenitor cell proliferation (21), but not for megakaryocyte maturation or platelet production (22, 23). MPL or THPO knockout mice appear hematologically normal except for thrombocytopenia but have decreased marrow HSC (24).In contrast, in human congenital amegakaryocytic thrombocytopenia (CAMT), MPL mutations, usually in the MPL distal extracellular cytokine receptor homology domain (CRHD) (25), cause thrombocytopenia, elevated plasma THPO, and severe marrow aplasia (26).

Importantly, the retrovirus MPLV, which caused an acute, fatal PV-like syndrome in mice and in vitro, also immortalized murine HPC, encodes an MPL gene with a truncated extracellular domain (27) (28), indicating that MPL is a proto-oncogene. Murine bone marrow cells expressing ectopic THPO caused a fatal, transplantable myeloproliferative disorder with splenomegaly, osteomyelofibrosis, pancytopenia, and leukemic transformation (29) (30). Conversely, murine marrow cells expressing ectopic EPO (31) or erythroid progenitor cells expressing JAK2 V617F caused erythrocytosis without significant EMH and did not engraft in secondary recipients (32), supporting a primary role for HSC, MPL, and THPO in MPN pathophysiology.

In humans, hereditary or somatic mutations that involve the MPL transmembrane domain or distal CRHD can have an ET or PMF phenotype (2), (33, 34). In addition, MPL bound by mutated *CALR* is inappropriately transported by this protein chaperone to the cell surface and activated, causing either an ET or PMF phenotype (35–37). Furthermore, germline single nucleotide polymorphisms (SNP) in the *MPL* distal CRHD that caused a variably penetrant, benign thrombocytosis phenotype with an elevated plasma THPO, occurred in specific ethnic groups (38, 39) and could be recapitulated in the mouse (40, 41). Hereditary THPO mutations causing uncontrolled THPO synthesis are associated with isolated thrombocytosis (42). In one family, however, such a THPO mutation was also associated with leukemic transformation or myelofibrosis (43).

Differing from *MPL* mutations, *JAK2* V617F causes PV, ET, and PMF. However, similarly to hereditary or somatic MPL (2) (38) as well as *CALR* mutations (35), impaired MPL cell-surface expression is a feature of *JAK2* V617F-positive PV, ET, and PMF (44, 45). How impaired expression of the hematopoietic growth factor receptor responsible for HSC survival, expansion and thrombopoiesis could lead to a myeloproliferative state has been a puzzle. In this review, we discuss how the unique dependence of HSC on the MPL–THPO axis together with the unusual pathophysiology of MPL in the MPN, creates a therapeutic target of opportunity to suppress MPN HSC while sparing normal HSC.

## Hematopoietic Stem Cell Physiology and Pathophysiology

Recent developments in HSC biology have reoriented our understanding of both HSC behavior in the bone marrow and their progression from an undifferentiated state to the committed HPC that give rise to erythrocytes, myeloid cells, and platelets. Importantly, HSCs require both the MPL–THPO axis and mature megakaryocytes to remain quiescent in their bone marrow niches (20, 46). The importance of localization of mature megakaryocytes to HSC bone marrow niches is such that a subpopulation of marrow HSC expresses the von Willebrand gene and gives rise directly to self-replicating megakaryocytic HSC (47). Other HSC-generated but lineage-restricted, self-replicating HSCs also give rise directly to megakaryocyte-erythroid and common myeloid repopulating stem cells, in addition to those progressing down the classical HSC pathway of commitment and differentiation (48).

The presence of megakaryocyte-primed HSC at the apex of the HSC hierarchy explains why thrombocytosis is a common presenting manifestation of an MPN. It also explains the mechanisms by which some ET patients only express *JAK2* V617F in their platelets (49), and why some *JAK2* V617F-positive PV patients only have erythrocytosis and thrombocytosis (50), in addition to those who have a complete panmyelopathy. Additionally, it provides an explanation for the transformation of ET to PV and PMF to PV and *vice versa* through HSC clonal succession (51).

This behavior must be distinguished from the natural history of MPN driver mutations to produce marrow fibrosis in PV and ET, because such behavior does not necessarily confer the same biologic characteristics as PMF (52). HSC behavior, of course, is not only MPN driver mutation-related, it is also strongly influenced by sex and age (53), and in PV, a proclivity to *JAK2* V617F homozygosity by uniparental disomy (54). Most importantly, however, the primacy of the MPL–THPO axis in HSC behavior not only under normal circumstances but in the MPN cannot be ignored from a therapeutic perspective.

## MPL Physiology and Pathophysiology

The thrombopoietin receptor, MPL, is a member of the type 1 homodimeric cytokine receptor family together with the EPO, prolactin, growth hormone, and granulocyte-colony stimulating factor receptors. Among this receptor family, MPL is unique because it has a reduplicated extracellular CRHD, of which, the distal CRHD is the site of THPO binding. The distal CRHD is also the site of germline SNP causing benign familial thrombocytosis (38, 39), a hot spot for the mutations causing CAMT (25) as well as the site of terminal sialylation during receptor maturation, which is impaired in the MPN (55). Like the other hematopoietic growth factor receptors, MPL is responsible for the metabolism of its cognate ligand, but in contrast to the other hematopoietic growth factors, the production of which is mainly regulated by demand, THPO is constitutively produced in the liver (56), and production of a critical platelet mass is required to maintain a constant platelet count (57).

The observation that MPL cell-surface expression was impaired in JAK2 V617F-positive PV, PMF (44) and ET (45) appeared counterintuitive since MPL is the sole hematopoietic growth factor receptor expressed in HSC, and the MPNs are diseases with autonomous myeloproliferation (58). Additionally, impaired MPL cell-surface expression was universal in the MPN since it also occurred with *MPL* (59, 60) and *CALR* (35) mutations, and in familial thrombocytosis due to germline MPL SNP in the MPL distal CRHD as well (38, 39). Mechanisms for impaired MPL cell-surface expression include *MPL*, *CALR* or *JAK2* mutations, increased MPL turnover, or impaired post-translational processing, and in PMF, reduced GATA1 expression, impaired megakaryocyte differentiation, an associated ribosomal deficiency state and impaired megakaryocyte-specific protein expression (61).

CAMT is due to *MPL* mutations (25), primarily in the distal CRHD; all three of the above mechanisms are responsible for impaired MPN MPL cell-surface expression (2, 33, 55, 62). Germline SNPs causing impaired MPL cell-surface expression are also located in the distal CRHD and appear to be due to impaired post-translational processing (38, 39). MPL is produced as an incompletely-glycosylated 80 kDa protein, which is fully glycosylated in the Golgi to a 95 kDa mature protein with JAK2 as its obligate chaperone (17). Normally, both incompletely glycosylated and mature MPL proteins are expressed at the cell-surface and both are responsive to THPO-induced signaling (62, 63). While MPN driver mutations and *MPL germline* SNP result in impaired terminal MPL sialylation in the distal CRHD (55), *JAK2* V617F activation adds an additional defect. JAK2 is responsible for enhancing MPL stability and recycling (17, 61), but *JAK2* V617F also increases MPL ubiquitination and proteasomal degradation, leading to decreased MPL recycling and half-life, predominantly involving mature MPL (61).

Importantly, since megakaryocytes do not require THPO for megakaryocyte maturation or platelet production, impaired MPL cell-surface expression does not affect these processes (20, 21). It does, however, impair plasma THPO clearance by megakaryocytes and platelets (57). This leads to increased plasma THPO levels in the MPN (64, 65) and a continuous signal for HPC to proliferate, either collectively (*MPL* S505N, *MPL W515* K/L, *CALR del/+* and *JAK2* V617F), or limited to megakaryopoiesis alone (*MPL* K39N and *MPL* P106L), because adequate cell-surface MPL is still expressed in HCP for this purpose (66). Importantly, the MPN phenotype partially emulates that of wild-type mice and humans with unregulated THPO production (42), which is reversible in mice by inhibition of THPO synthesis (30).

Mouse hematopoiesis, of course, differs from human hematopoiesis because in the mouse, the platelet count is approximately three times the human platelet count. The mechanism for this appears to be due to the presence of an asparagine at amino acid residue 39 in the mouse MPL distal CRHD, which is otherwise highly homologous to the human MPL distal CRHD. Importantly, in humans with the germline SNP *MPL* K39N, where asparagine is substituted for lysine [(38)], MPL expression is impaired and affected individuals’ phenocopy mouse hematopoiesis with thrombocytosis and an elevated plasma THPO, suggesting a human biologic model for MPN pathophysiology.

## Role of Thrombopoietin in Myeloproliferative Neoplasm Pathophysiology

Because MPL is essential for HSC quiescence and survival in the marrow osteoblastic niche (22, 23, 67) and is also responsible for THPO catabolism (57), we hypothesized that impaired MPL cell-surface expression was essential for MPN phenotypic behavior, causing unregulated HPC proliferation and, eventually, myelofibrosis due to increased plasma THPO (30), depending on the MPN driver mutation allele burden, while also causing HSC loss from the marrow (22).

**Figure 1:**
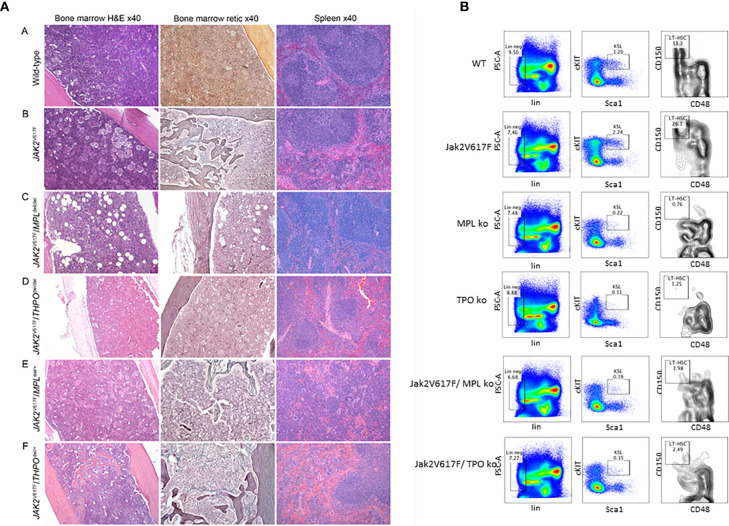
**(A)** *MPL* and *THPO* knockout (del/del) genotypes mitigate marrow and spleen histopathology in a *JAK2* V617F transgenic mouse model of PV but this was restored in *JAK2* V617F*/THPO* del/+ mice. Representative marrow and spleen histology at >33 weeks in wild-type mice and *JAK2* V617F, *JAK2* V617F*/MPL* del/del *JAK2* V617F*/THPO* del/del, *JAK2* V617F*/MPL* del/+, and *JAK2* V617F*/THPO* del/+ transgenic mice. All images were taken with Zeiss AX10 Imager microscope using a Plano-APO 10×, 0.45 NA lens with a tungsten 3,200 K light source. The imaging medium was digital photomicrography using a bright field microscope and a Pro Res 14 camera with Adobe Photoshop CC acquisition software. Magnification was 40× for all images (69). **(B)** *MPL* and *THPO* knockout genotypes reduce the LT-HSC (CD150+CD48-) population in a *JAK2* V617F transgenic mouse model of PV. Flow cytometry of marrow LT-HSC at >16 weeks in wild-type (n = 4), *MPL* knockout (n = 3), and *THPO* knockout (n = 3) mice and *JAK2* V617 (n = 4), *JAK2* V617F*/MPL* knockout (n = 3) and *JAK2* V617F*/THPO* knockout (n = 3) transgenic mice. The marrow LT-HSC population was 13% of the total LSK population in wild-type mice, 26% of the total LSK population in *JAK2* V617F transgenic mice and 0.76 and 1.25% respectively in the *MPL* knockout and *THPO* knockout mice and 1.98 and 2.08% respectively of the total LSK population in *JAK2* V617F*/MPL* knockout and *JAK2* V617F*/THPO* knockout transgenic mice. WT *vs JAK2* V617F, P <0.029; *MPL* knockout *vs JAK2*V 617F, P *<* 0.022; *THPO* knockout *vs JAK2* V617F, P *<* 0.021; *JAK2* V617F *vs JAK2* V617F*/MPL* knockout, P *<* 0.023; *JAK2* V617F *vs JAK2* V617F*/THPO* knockout, P *<* 0.034. The number (n) of mice of each genotype studied is in parentheses (69).

To test this, we employed a *JAK2* V617F transgenic mouse model, which mirrors PV natural history, with erythrocytosis, granulocytosis, thrombocytosis, splenomegaly and eventually anemia and osteomyelofibrosis (68). Unsurprisingly, when crossed with an MPL knockout mouse, there was abolition of the PV histologic phenotype (**Figure 1A**) and a marked decrease in marrow HSC (**Figure 1B**), which could only be partially alleviated with expression of one MPL allele. This confirmed an essential role for MPL in this transgenic mouse model of PV (69).

PV transgenic mice differed from human PV because the plasma THPO level was reduced, not increased, suggesting that the lower plasma THPO in contrast to wild-type mice was due to increased THPO utilization by the *JAK2* V617F-mediated increase in the megakaryocyte and platelet pools, a characteristic also found in mouse models with *MPL* (59) or *CALR* mutations (70).

To study the role of THPO in the *JAK2* V617F transgenic mouse phenotype, we crossed this mouse with a THPO knockout mouse. Unexpectedly, the PV phenotype was modified; this involved both the reversal of splenomegaly and osteomyelofibrosis, and a reduction in marrow HSC, despite the biallelic expression of functional MPL expressing *JAK2* V617F. Restoration of one THPO allele completely restored the PV phenotype, in contrast to the incomplete restoration of the PV phenotype with a single *MPL* gene (*JAK2* V617/*MPL* del/+). These results indicate that constitutive MPL signaling through *JAK2* V617F alone was not sufficient to support the full PV phenotype in this transgenic mouse model.

Our observation that *THPO* gene deletion abrogated the PV phenotype in a *JAK2* V617F transgenic mouse model differs from the observations of Sangkhae et al. (71). Their study employed a *JAK2* V617F transgenic mouse model with an ET phenotype (72) and only 16 weeks of observation compared to our study, making their results not comparable with our *JAK2* V617F transgenic mouse model, which recapitulated the natural history of PV, and required over 33 weeks of observation for full expression of the disease phenotype. Moreover, although Sangkhae et al. claimed that THPO was not necessary for expression of the ET phenotype in their mouse model, in agreement with our results, in their *JAK2* V617F/*THPO* knockout transgenic mice, thrombocytosis was eliminated and *in vitro* HPC proliferation, megakaryocyte number and size, and spleen size were also reduced, indicating THPO dependence in their *JAK2* V617F ET transgenic mouse model. Importantly, in both transgenic mouse models, abrogation of THPO production did not reduce blood counts below the baseline levels of control mice (68, 70).

Our observation that the MPNs are hematopoietic growth factor-dependent disorders, particularly in the *JAK2* V671F heterozygous state is also supported by *in vitro* studies. For instance, human PV BFU-E heterozygous for *JAK2* V617F responded *in vitro* to erythropoietin like normal BFU-E (13). Similarly, MPN HPC hematopoietic growth factor-responsiveness was observed *in vitro* with *MPL* (55) and *CALR* (4, 37) mutations.

Reduction in marrow HSC in the absence of the *MPL* or *THPO* genes in our study also strongly supports our assertion that the MPNs are hematopoietic growth factor-dependent-diseases. Significantly, a small molecule antagonist of MPL preferentially inhibited *JAK2* V617F-positive proliferation of PV HSC both *in vitro* and *in vivo* compared to normal HSC (73), while *in vitro*, PV HPC and murine cell lines expressing *MPL* (55)] or *CALR* (4, 37) mutations were still THPO-responsive despite the presence of constitutively-activated JAK2.

Furthermore, treatment of wild-type mice with an MPL antagonist antibody allowed non-myeloablative bone marrow transplantation, verifying the requirement for THPO to maintain HSC in their marrow niches (23). Importantly, while the absence of functional MPL causes CAMT in humans, MPL cell-surface expression is impaired, but not absent in MPN HSC and HPC, which, as demonstrated experimentally (59), should give normal HSC and HCP a survival advantage when exposed to a THPO antagonist.

## Control of Thrombopoietin Production as A Treatment for the Myeloproliferative Neoplasm

**Figure 2:**
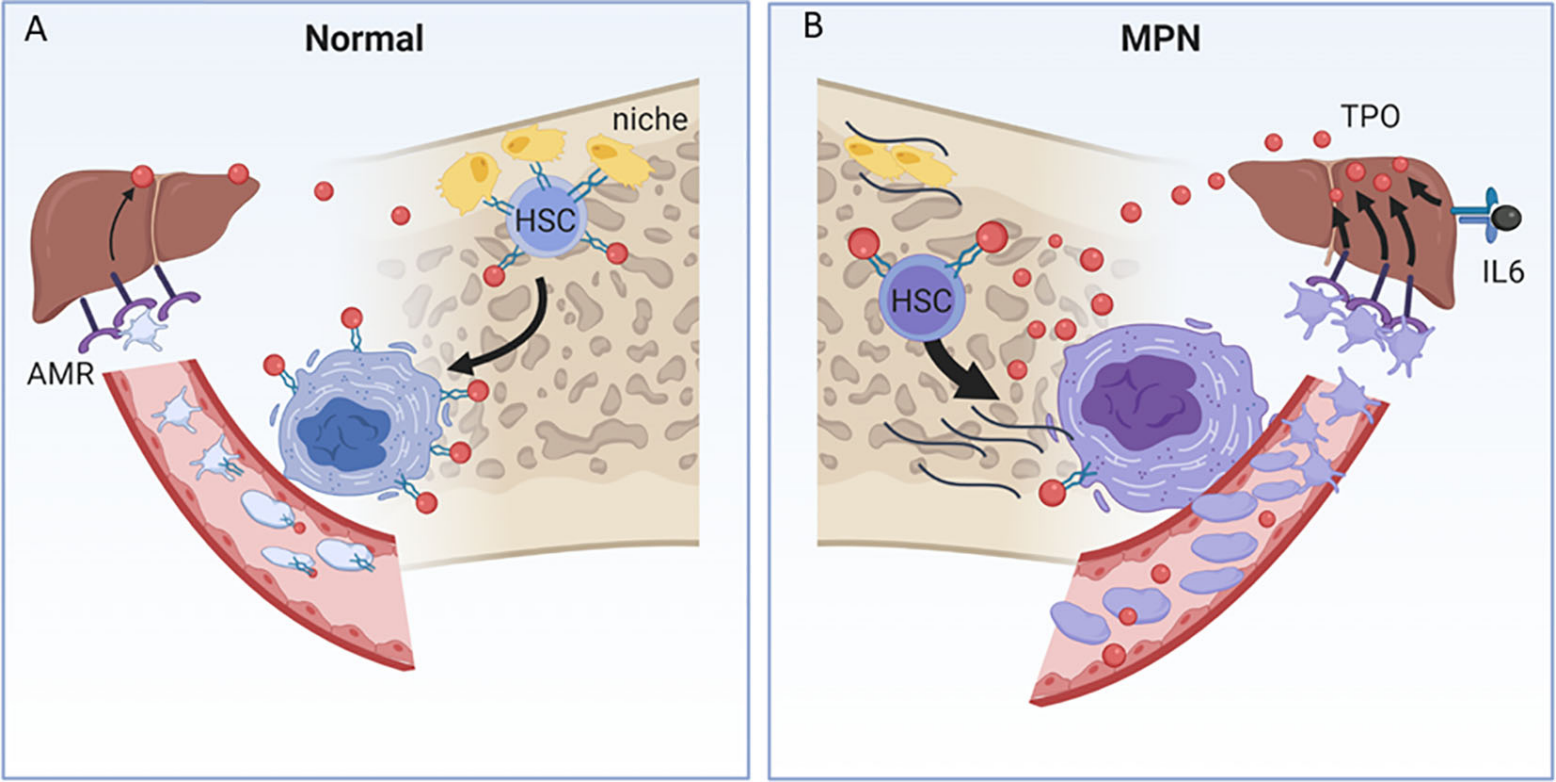
**(A)** THPO metabolism in normal and MPN contexts. **(A)** In the endosteal niche, HSCs are tethered to osteoblasts (yellow cells) by adhesive proteins and receptors, including THPO (red ball) and its receptor, MPL (green receptor), and are maintained in a quiescent state. MPL also clears THPO from the circulation by binding and internalizing THPO in megakaryocytes and platelets. Desialyated platelets bind to the Ashwell–Morell receptor (AMR) in the liver, and induce hepatic THPO production, in addition to the constitutive hepatic production of THPO. **(B)** In the MPN, loss of MPL surface expression and increased THPO/MPL/JAK/STAT signal transduction promote HSC untethering and egress from the endosteal niche to the sinusoidal niche. Mutant, hypersignaling MPN HSCs and megakaryocytes produce inflammatory cytokines, which alter the marrow cellular microenvironment, suppress normal hematopoietic stem cells and promote myelofibrosis (black lines). Loss of MPL cell-surface expression in megakaryocytes and platelets results in the inability to clear plasma THPO, further providing a source of THPO for HSC and megakaryocyte hyperstimulation. MPN platelets are both increased and are more avidly bound to the AMR, which further enhances THPO production. Interleukin-6, produced by the MPN inflammatory milieu, independently enhances hepatic THPO production.

Current models of HSC indicate that they remain quiescent in their bone marrow niches anchored to osteoblasts by a number of adhesive molecules as well as by THPO (**Figure 2A**). Recent studies, however, suggest that the liver is the primary source of THPO and that osteoblast THPO is not required. In addition, while hepatic THPO production is constitutive, recent studies have identified an important regulatory function of platelet clearance through the Ashwell–Morrell receptor (AMR) (74) (**Figure 2A**). Hepatic THPO is also markedly increased in inflammatory conditions and is attributed to the direct action of interleukin-6 on hepatic THPO production (75).

THPO metabolism is markedly altered in the MPN, both due to reduced clearance and enhanced production (**Figure 2B**). Loss of MPL surface expression in megakaryocytes and platelets, in addition to untethering HSCs from their niche, results in loss of THPO clearance and higher THPO levels that feed back to enhance HSC MPL/JAK/STAT signal transduction in both normal and mutant clonal contexts and drives pathologic myelofibrosis and inflammatory cytokine pathways (44, 76, 77) (**Figure 2B**). The MPN context is also one of enhanced production of hepatic THPO, both through increased MPN platelet clearance through the AMR, and through IL-6 mediated hepatic stimulation associated with high inflammatory milieu in the MPN (77, 78) (**Figure 2B**).

Thus, in addition to inhibiting the MPL–THPO interaction at the level of the HSC, antagonizing hepatic THPO production could be a feasible, target-specific, non-myelotoxic therapeutic approach. Gene silencing of hepatic targets can be achieved by using the AMR as entry for modified RNA inference into hepatocytes, and is a safe and effective therapy in human blood diseases (79). Recently, THPO has been effectively silenced in murine models and is effective in lowering THPO levels and platelet counts using this basic technology (80, 81).

## Conclusions

Based on our observations, we propose that impaired MPN MPL cell-surface expression leads to inappropriately high plasma THPO because of failure of THPO clearance by MPN platelets and megakaryocytes. Increased plasma THPO enhances activated JAK2 signaling in HPC, while impaired MPL cell-surface expression weakens the ability of HSC to remain in the marrow osteoblastic niche. Eventually, marrow HSC loss due to *in situ* differentiation or migration from the marrow and sequestration in the spleen, together with continued megakaryocyte stimulation by the elevated plasma THPO, produces a PMF phenotype, regardless of the MPN driver mutation. Viewed from this perspective, the MPNs are in part hematopoietic growth factor-dependent disorders, and targeting the MPL–THPO axis by a THPO antagonist or suppressing hepatic THPO production could be an effective, non-myelotoxic therapeutic strategy.

## Author Contributions

Both authors planned and wrote the manuscript. All authors contributed to the article and approved the submitted version.

## Funding

JLS, National Cancer Institute CA108671; ARM, Myeloproliferative Research Foundation Challenge Grant; JS and AM, National Heart, Lung and Blood Institute HL145750. The funders had no role in study design, data collection and analysis, decision to publish, or preparation of the manuscript.

## Conflict of Interest

The authors declare that the research was conducted in the absence of any commercial or financial relationships that could be construed as a potential conflict of interest.
